# Recent Advances for Flame Retardancy of Textiles Based on Phosphorus Chemistry

**DOI:** 10.3390/polym8090319

**Published:** 2016-08-25

**Authors:** Khalifah A. Salmeia, Sabyasachi Gaan, Giulio Malucelli

**Affiliations:** 1 Additives and Chemistry, Advanced Fibers, Empa, Swiss Federal Laboratories for Materials Science and Technology, St. Gallen CH-9014, Switzerland; sabyasachi.gaan@empa.ch; 2 Department of Applied Science and Technology, Local INSTM Unit, Politecnico di Torino, Viale T. Michel 5, 15121 Alessandria, Italy

**Keywords:** flame retardants, fibers and fabrics, phosphorus-based products, biomacromolecules, cotton, polyester, cotton-polyester blends, polyamide

## Abstract

This paper aims at updating the progress on the phosphorus-based flame retardants specifically designed and developed for fibers and fabrics (particularly referring to cotton, polyester and their blends) over the last five years. Indeed, as clearly depicted by Horrocks in a recent review, the world of flame retardants for textiles is still experiencing some changes that are focused on topics like the improvement of its effectiveness and the replacement of toxic chemical products with counterparts that have low environmental impact and, hence, are more sustainable. In this context, phosphorus-based compounds play a key role and may lead, possibly in combination with silicon- or nitrogen-containing structures, to the design of new, efficient flame retardants for fibers and fabrics. Therefore, this review thoroughly describes the advances and the potentialities offered by the phosphorus-based products recently developed at a lab-scale, highlighting the current limitations, open challenges and some perspectives toward their possible exploitation at a larger scale.

## 1. Introduction

Textiles play an important role in everyday life: one of their main drawbacks refers to their structure, as they are mainly made of organic polymers, which conversely, if not inherently flame-retarded (such as polyaramides and polyphosphonate fibers), are flammable and potentially dangerous species. Specifically referring to fibers and fabrics, the annual UK fire statistics have clearly demonstrated that most of fire incidents occur in houses, involving upholstering furniture, bedding and nightwear [1].

In this context, the ease of flammability of textiles has been faced by designing and synthesizing suitable flame retardants (FRs), i.e., additives that are able to suppress or delay the appearance of a flame and/or reducing the flame-spread rate (flame retardants) or delaying ignition or reducing the rate of combustion when needed (fire retardants) [2].

**Figure 1:**
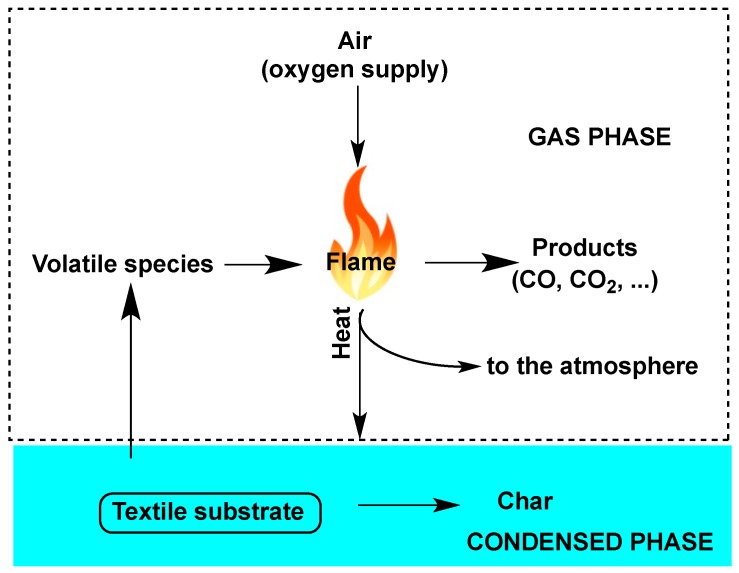
Scheme of the textile combustion cycle.

From an overall point of view, combustion in the presence of flames is a gas-phase process exploiting the oxygen taken from the surroundings. As a consequence, before the occurrence of the combustion process, the textile undergoes degradation: some of the so-obtained degradation products turn into combustible volatile species that, in combination with oxygen, fuel the flame. If the heat generated in the combustion is sufficient, it can be easily transferred to the textile substrate, hence giving rise to further degradation phenomena and supporting a self-sustaining combustion cycle (Figure 1).

On the basis of the chemical composition and their thermal and fire characteristics, if they are not inherently flame retarded, fibers and fabrics have to be treated with additives that may contain halogen, nitrogen, phosphorus, sulphur, boron, metals, etc., hence becoming flame retarded. The aforementioned additives can be added during spinning processes performed on synthetic fibers, or deposited on the synthetic or natural fiber/fabric surface, hence creating a protective layer/coating. Both finishing and coating methods can be exploited: concerning the former, the fiber/fabric is impregnated with a solution or a stable suspension that contains the FR additive. Conversely, the coating method involves the application of a continuous or discontinuous layer/film on both surfaces of the textile.

From an overall point of view, the self-sustaining combustion cycle of Figure 1 can be broken in the presence of FR additives, thus achieving the extinction of the flame or reducing the burn rate. In particular, they can:

- Lower the developed heat to below that necessary to carry on the combustion process.
- Modify the pyrolysis process to lower the quantity of flammable volatiles developed, favoring, at the same time, the creation the char, i.e., a carbonaceous residue that also limits the heat and mass transfer between the textile material and the flame.
- Isolate the flame from the oxygen/air supply.
- Release flame inhibitors (like chlorinated and brominated compounds) when the textile approaches the ignition temperature.
- Lower the heat flow back to the fabric, hence limiting or preventing further pyrolysis.
- Favor the formation of a char or an intumescent protective layer when the textile interacts with a flame or a heat source.

A review published in 2011 succeeded in summarizing the state of the art for the different commercially available flame retardants for textile materials, which were classified according to the following periods [3]:

- 1950–1980: The “golden period” of flame retardant research, involving the appearance of the first patents on FRs based on organophosphorus compounds for cellulosic textiles (i.e., cotton). During this period, inherently FR synthetic fibers bearing aromatic structures were also developed.
- 1980–late 1990s: Very limited advances of the research in FRs were achieved during this period.
- 2000 onward: Several efforts were carried out in the design of char-former flame retardant additives, possibly containing phosphorus-based products. Another goal was the investigation into the possibility of replacing bromine derivatives with other less toxic and efficient products. Furthermore, during this period, nanotechnology was demonstrated to show outstanding potential for conferring flame retardant features to fibers and fabrics, through the use of nanoparticles having different aspect ratios. In particular, the exploitation of both top-down (using preformed nanoparticle suspensions) and bottom-up (exploiting the generation of single nanoparticles or nanoparticle assemblies—even hybrid organic-inorganic structures) was successfully considered [4,5].

Specifically referring to the textile field, FRs can also be classified according to their “laundry durability”: indeed, a non-durable FR is washed off immediately when soaked in water, but may resist dry cleaning. Conversely, semi-durable FRs are able to resist water-soaking and possibly a few washes, while durable FRs endure some 50 or 100 washing cycles.

However, according to the very stringent directives recently promoted by the EU community and USA, some of the halogenated compounds (such as brominated diphenyl derivatives) have been banned, as they have clearly shown a high toxicity for both animals and humans [6].

The aforementioned disadvantages stimulated the scientific community toward the design and development of phosphorus-based compounds, which seem less toxic and may represent a suitable alternative to their halogen-based counterparts. Though it is not a general case that all phosphorus compounds are non-toxic, the development of new flame retardants based on phosphorus compounds has shown that they have lower toxicity profiles as compared to halogen-based counterparts [7,8]. In general, the development of any new flame retardant should involve a complete assessment of its performance in material as well as its toxicity.

In this context, some new products have been designed and nowadays are commercially available. In particular, Trevira CS^®^, which is based on the use of a phosphorus-containing comonomer (in the form of propionylmethylphosphinate), has been exploited for conferring flame retardant properties to polyester fibers and fabrics [9,10]. Regarding cotton and cellulosic-rich substrates, the present focus is either on the synthesis of effective non-halogenated additives for coatings and back-coated fabrics or on the utilization of hydroxymethylphosphonium salts (Proban^®^) or N-methylol phosphonopropionamide derivatives (Pyrovatex^®^).

The chemistry of the Proban^®^ process exploits a tetrakis(hydroxymethyl) phosphonium–urea condensate, which, after padding, is crosslinked by ammonia gas in a dedicated plant and then subjected to peroxide oxidation for stabilizing the resulting polymeric matrix [11]. The washing fastness of this treatment is due to the deposition of the chemical within the fibers by a construction of a polymer network during the heating process: as a consequence, Proban^®^ is not linked to the fibers but is mechanically retained within the fiber interstices. One of its major disadvantages refers to the possible release of formaldehyde during the fabric use [12].

Conversely, the chemistry behind Pyrovatex^®^ is based on a conventional pad-dry-cure process in the presence of a methylolated crosslinking agent, which is responsible for the formation of covalent bonds with the hydroxyl groups of the cellulosic substrate. Nonetheless, about 50% of Pyrovatex^®^ FR treatment has been reported to be lost during the first laundry occurrence, because of the extraction of unreacted products, though it remains stably linked thereafter.

Despite the efficiency of such commercially available treatments, both academic and industrial researchers are still seeking worthy alternatives, also taking into account the requirements that have to be fulfilled by the new products. In particular, with respect to the already existing FRs:

- Any new flame retardant should show equivalent or superior ease of application.
- Any new flame retardant should not emit formaldehyde during application or service.
- Any new flame retardant should provide comparable textile service-life features, specifically referring to durability, comfort, tensile properties, outward appearance and aesthetics.
- Any new flame retardant should possess an overall comparable cost-effectiveness to the already existing chemicals and preferably be less costly.
- Any new flame retardant should possess equivalent or even lower toxicity and environmental impact.
- Air permeability of the treated textiles should be maintained, irrespective of the possible high amounts of chemicals needed to provide flame retardant features.
- Any new flame retardant should not cause any alteration in the hue of the dye and/or dyeability of the fibers/fabrics.

Nowadays, the approach adopted by the scientific community is being slightly changed: indeed, the durability of any new flame retardant is still needed, but the novel processes and methods developed in last five years seem to be more addressed to the design of low impact and eco-friendly systems.

In this context, the present paper aims at providing an overview of the recent advances in the design of phosphorus-based FRs, also in combination with nitrogen- or silicon-containing structures, for different fibers and fabrics: in particular, the evolution from chemical to low environmental impact products will be thoroughly described, highlighting the current achievements and limitations, as well as the open challenges and perspectives.

## 2. Assessment of the Fire Behavior of Textiles

This section summarizes the current methods that allow assessing the reaction of a textile toward the exposure to a flame or a heat flux. All the developed methods take into account that, for textiles, the high fiber surface to mass ratio favors their easy ignition; in fact, these materials burn faster than other bulk polymers.

Generally speaking, ignition occurs when a small flame is applied to flammable fabrics for no more than 12 s and the textile continues to burn after the removal of the flame. Therefore, most of the work on flammable fabrics focuses on the evaluation of the facility of ignition, the rate and extent of flame spread, the duration of flame propagation, the heat release and heat of combustion. All these parameters are merged with a quantitative portrayal of burning wreckages, such as melt dripping. It is very difficult to find a single test method able to measure all the aforementioned parameters. Concerning the fabrics that show self-extinction, such as flame-retarded fabrics, tests comprise the evaluation of time of afterflame and afterglow and extent of fire damage (specifically referring to char length, dimension of holes, or damaged sample length).

The measurement of textile flammability involves either scientific (i.e., research) test or the standard test methods. The former provide information suitable for assessing the burning behavior and are exploited for the design of new FRs or fire-retardant treatments.

Limiting oxygen index (LOI), also called oxygen index (OI), is one of the most popular scientific methods, used in many standards, such as ISO 4589 and ASTM D2863. LOI denotes the minimum concentration (vol %) of O_2_ in a mixture of O_2_ and N_2_ that will just sustain flaming combustion of a material in a candle-like manner. Textile materials burn rapidly when they exhibit LOI values up to 21 vol %, while they burn slowly when LOI is in between 21 vol % and 25 vol %. LOI values beyond 26 vol % indicate some flame retardant features [13].

The obtained LOI values may be affected by several fabric structural parameters, when measured for the same fiber type: this makes LOI values relative and not absolute data. In addition, the textile material is ignited at the top and thus it burns vertically downward (i.e., in candle-like manner) which is opposite to the burning of any material freely suspended.

Simple ignition tests (used in many standards, such as BS 5438 and EN ISO 6941) represent another usual approach for assessing the flammability of a textile material: more specifically, a standard gas flame is applied to the face or lower edge of a vertically oriented fabric sample; ignition is examined by visual observations and the time needed to ignite the specimen is recorded. The textile does not pass the test when, after the removal of ignition source, the flame achieves any end of the sample. If the flame reaches extinction, the char length, dimension of holes, afterglow, and type of any wreckage (molten drops, etc.) are thoroughly evaluated.

Flame spread (UL-94, which contains EN 60695 11-10, ASTM D 635-03 and D 3801-00) is a bench-scale test which measures the rate of flame spread usually calculated as the ratio of the distance to the time taken of the advancing flame front to reach defined distances marked on the fabric specimen. The upward fire spread is far faster than downward and horizontal flame spread and, hence, adopted as a better means of assessing the fire hazard of a fabric.

Although not specifically designed for fabrics, cone calorimetry tests (according to ISO 5660) have become a standard bench scale model of early flaming [14,15]. In particular, the cone calorimeter mimics the penetrative burning seen as fire burning into a specimen. It evaluates the heat release rate and the effective heat of combustion from a burning material exposed to a controlled radiant heat source (ISO 5660 part 1). Usually, such parameters as Time To Ignition (TTI), Total Heat Release (THR), Heat Release Rate and corresponding peak (pkHRR), Effective Heat of Combustion (EHC), Mass Loss (ML) and Mass Loss rate (MLR) can be evaluated. The cone calorimeter can also be utilized to evaluate smoke generation (ISO 5660 part 2): in this case, the measured parameters include the determination of CO and CO_2_ concentrations, as well as the assessment of smoke density (Specific Extinction Area–SEA, Total Smoke Production–TSP, etc.).

The micro-combustion calorimeter (MCC) has recently been standardized (ASTM D7309-13) and exploited for evaluating the flammability of polymers [16,17]. In this process, a small specimen (about 2–10 mg) undergoes pyrolysis through a fast heating up in inert atmosphere (with a heating rate below 1 °C/s). The obtained pyrolysed products are then mixed with O_2_/N_2_ mixture to expedite combustion. The oxygen concentration and flow rates of the combustion gases are evaluated, and the amount of generated heat is calculated on the basis of oxygen consumption calorimetry.

## 3. Phosphorus Chemistry in FRs: An Overview

**Figure 2:**
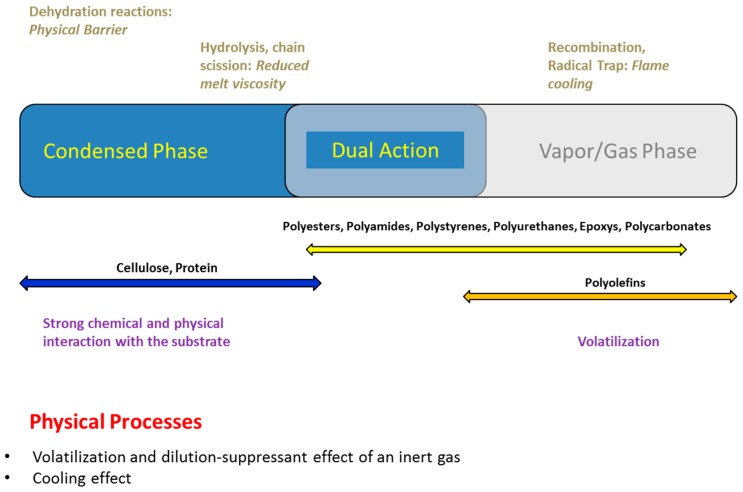
Mode of action of phosphorus-based flame retardants.

Phosphorus-based flame retardants are quite versatile in their flame retardant action. Phosphorus compounds often exhibit both condensed and gas phase activity [18]. A simplified scheme of various flame retardant actions of phosphorus is presented in Figure 2.

An additive is considered to be active in condensed phase if it alters the thermal decomposition characteristics of the polymer by a chemical reaction. Hydrolysis, dehydration, chain scission or de-polymerization are some of the main chemical reactions occurring in condensed phase activity. This activity is usually characterized by a reduction in the decomposition temperature of the polymer and increased formation of char residue at elevated temperatures [19].

In some cases, the de-polymerization of thermoplastic polymer chains in the presence of a heat source reduces the viscosity of the system and enables it to retreat from the fire without producing any residue.

The efficiency of phosphorus compounds to change the decomposition and combustion characteristics of polymers makes their fire suppressant use imperative. Depending on the substrate and their chemistry, there could be chemical interactions in the condensed phase at elevated temperatures, which lead to changes in the decomposition pathway of the polymer and possible formation of carbonaceous char residues on the surface of decomposing polymer, hence preventing its further oxidation. In other instances, the phosphorus compounds and some of their decomposed products preferably volatilize from the polymer substrate when heated. These phosphorus species further decompose to release reactive phosphorus species, which then interact with the combustion intermediates in the gas phase as inhibitors. In most cases, such interactions lead to recombination of the H and OH radicals and prevent their oxidation.

The condensed or gas phase activity of phosphorus compounds significantly depends on their structure, as well as on the polymer substrate. For example, in case of natural polymers like cellulose and wool, the phosphorus compounds primarily exhibit condensed phase activity where dehydration of the polymer, leading to the formation of a thermally stable char, is the predominant mechanism. Referring to synthetic polymers containing oxygen and nitrogen atoms in their structure, catalytic hydrolysis of the ester or amide groups by phosphorus acids promotes an enhanced melt dripping and fast shrinkage from flame. As far as olefin-based polymers are considered, the phosphorus compounds mainly act in the gas phase by recombining the key fuel species such as H and OH radicals and preventing their oxidation. Some minor physical effects due to volatilization of phosphorus compounds and dilution of the fuel can also occur.

## 4. Chemical Phosphorus-Based FRs for Textiles

The following paragraphs will describe the recent advances concerning the use of phosphorus-based chemical products, suitable for conferring flame retardant properties to different fibers and fabrics. In general, Table 1 summarizes the recent findings of P-based flame retardants and their performance on textile fabrics.

### 4.1. Dioxaphosphorinane Derivatives for PET Fibers

**Figure 3:**
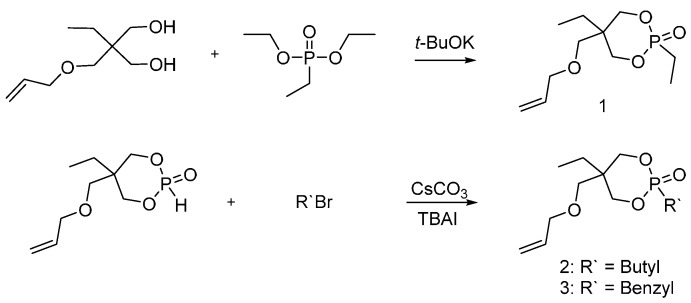
Representative synthesis of monomers (**1**), (**2**) and (**3**).

These new flame retardants were designed to exhibit similar performances in their activity to Antiblaze 19^®^, i.e., a trimethylolpropane methylphosphonate oligomer obtained from the reaction of trimethylolpropane phosphite with dimethyl methylphosphonate [54], employed for poly(ethyleneterephtalate) (PET) fibers. In particular, Negrell-Guirao and co-workers [20] synthesized 2,5-ethyl-5-(allyloxymethyl)-2-oxo-1,3,2-dioxaphosphorinane (**1**), 2-butyl-5-ethyl-5-(allyloxymethyl)-2-oxo-1,3,2-dioxaphosphorinane (**2**) and 2-benzyl-5-ethyl-5(allyloxymethyl)-2-oxo-1,3,2-dioxaphosphorinane (**3**) (Figure 3).

**Figure 4:**
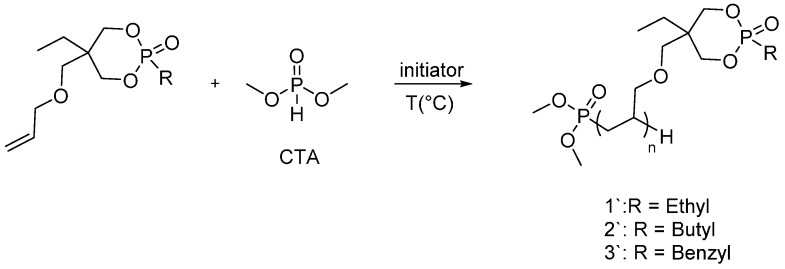
Radical polymerization of dioxaphosphorinane monomers, using dimethyl phosphite as CTA.

Due to the two pseudo-asymmetric centers for dioxaphosphorinane monomers, compounds (**1**), (**2**) and (**3**) exist as diasterioisomers. However, the radical polymerization of dioxaphosphorinane monomers shows the influence of the presence of a chain transfer agent (CTA) on the efficiency of the radical polymerization reaction. Moreover, dimethyl phosphite can play a role by enhancing the fire retardant efficacy of dioxaphosphorinane derivatives. As the CTA concentration increases, the monomer conversion increases as well and the degree of polymerization decreases (Figure 4).

The products of the polymerization reaction were isolated as mono- and di-adduct, rather than high *M*_W_ polymers. Furthermore, the flame retardancy of these dioxaphosphorinane derivatives was not assessed: therefore, it was not possible to make a real comparison with Antiblaze 19^®^.

### 4.2. UV-Curable Flame Retardants Coatings

**Figure 5:**
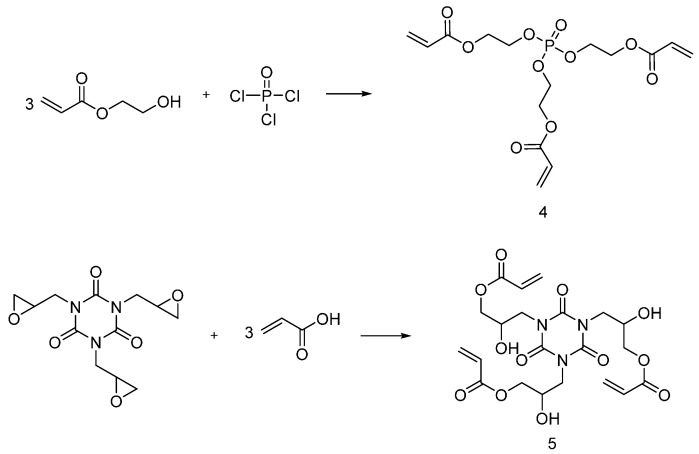
Schematic synthesis of UV-curable monomers (**4**) and (**5**).

Xing and co-workers designed and applied UV-curable flame retardants coatings able to protect cotton fabrics from heat penetration and flame spread [21]. In particular, as shown in Figure 5, tri (acryloyloxyethyl) phosphate (**4**) and triglycidyl isocyanurate acrylate (**5**) were synthesized and employed for impregnating cotton fabrics, using 1:1 weight ratio of the two monomers in acetone solution with 0.05 g/mL, 0.10 g/mL and 0.20 g/mL of reactive compounds. The monomers were then cured using 4 wt % of photoinitiator under a UV-lamp.

The formation of the UV-cured flame retardant coatings on the cotton surface was confirmed using Attenuated Total Reflectance (ATR) and Scanning Electron Microscopy (SEM) analyses. The flame retardancy of the treated fabrics was also assessed: in particular, LOI values were found to increase with increases in the flame retardants’ content. A similar trend was also noticed from Micro-scale Combustion Calorimetry measurements. Furthermore, cotton fabrics treated with flame retardant monomers (**4**) and (**5**) showed good durability to laundry. The MCC results after laundering showed a slight increase in Heat Release Capacity and Total Hydrocarbons values, which, however, were still lower than those of untreated cotton. In general, the UV-curable flame retardant coating based on monomers (**4**) and (**5**) showed condensed phase mode action as confirmed by the increased char formation of the treated cotton.

**Figure 6:**
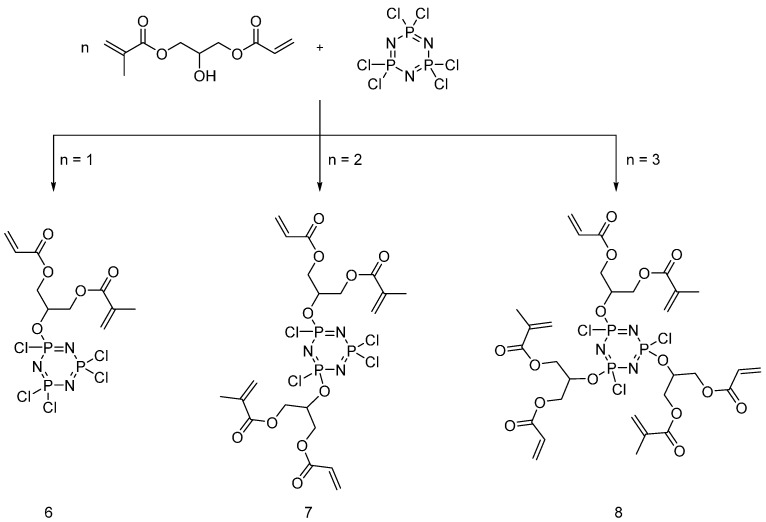
Representative schematic synthesis of UV-curable monomers (**6**), (**7**) and (**8**).

Very recently, Edwards et al. [22] synthesized novel UV-curable flame retardants obtained from the reaction of hexachlorotriphosphazene, in the presence of triethylamine, with different molar equivalents of 1-(acry-loyloxy)-3-(methacryloyloxy)-2-propanol, affording monomers (**6**), (**7**) and (**8**), (Figure 6).

The fabric samples were impregnated with UV-curable monomers in acetone solutions, containing different concentrations of monomers and photoinitiator. All three synthesized monomers showed an increase in coating yield with increases in the monomer concentration and UV exposure time; conversely, the photoinitiator concentration did not affect the coating yield. As far as flammability tests are considered, all the treated cotton samples showed enhanced flame retardancy with the formation of a coherent char. In particular, the higher phosphorus content in monomers (**6**) and (**7**) allowed achieving better fire performances as compared with monomer (**8**).

**Figure 7:**
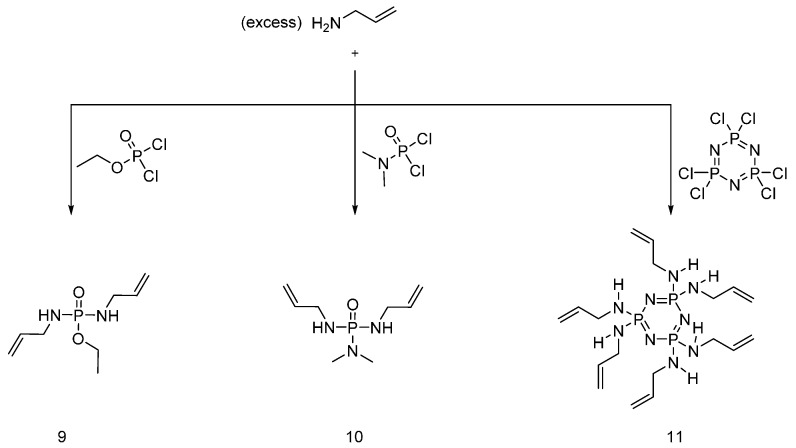
Representative schematic synthesis of UV-curable monomers (**9**), (**10**) and (**11**).

Pursuing this research, the same group [23] synthesized two new UV-curable monomers, namely di(allylamino)ethyl phosphate (**9**) and di(allylamino)dimethyl phosphoramide (**10**), and their flame retardant efficacy was compared to that of hexa(allylamino)cyclotriphosphazene (**11**) (Figure 7).

The obtained monomers were dissolved in acetone together with different amounts of photoinitiators and then applied onto cotton fabrics. It was found that monomers (**9**) and (**10**) were not suitable for UV-curing polymerization reactions: this finding was attributed to the fast evaporation of the monomers once they were exposed to UV radiation. Thus, cotton fabrics were impregnated these monomers and were tested without undergoing the UV-curing process. Although the treated fabrics did not burn when subjected to flame spread tests, hence revealing good flame retardant features, they could not be subjected to the UV-curing process.

**Figure 8:**
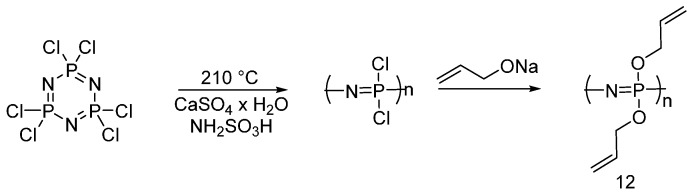
Synthesis of allyl-functionalized polyphosphazene (**12**).

In order to overcome this problem, Mayer-Gall and co-workers [24] synthesized allyl functionalized polyphosphazenes (**12**) starting from hexachlorotriphosphazene (Figure 8).

The obtained product was then used for cotton and different cotton/polyester blended fabrics, using an impregnation method (1 mL/g of 25 wt % of a solution of monomer (**12**) in acetylacetone) followed by UV-grafting in inert atmosphere (argon). The treated fabrics exhibited good flame retardancy with char formation, hence, confirming a condensed phase mode of action of the designed flame retardant system. The UV-cured FR coatings showed also a good washing fastness.

**Figure 9:**
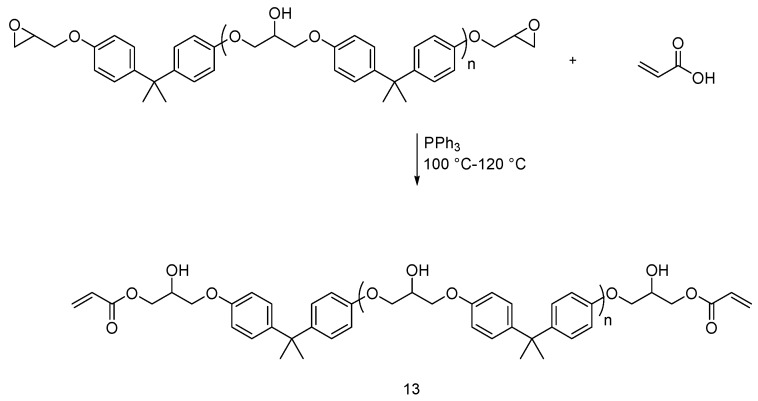
Schematic synthesis of oligomer (**13**).

Very recently, Yildiz et al. synthesized a UV-curable epoxy based oligomer adhesive formulation (**13**), by reacting acrylic acid with bisphenol-A-diglycidylether (DGEBA) [25]. The reaction is shown in Figure 9.

The system was employed to adhere cord fabrics (i.e., a polyester/polyamide blend) onto the rubber surfaces. Vinyl phosphonic acid was then incorporated into the epoxyacrylate-based adhesive at different weight ratios to enhance the flame retardancy of the treated fabrics; 3 wt % of photoinitiator in methyl ethyl ketone was also added. The fabrics were dip-coated and the solution was then homogeneously spread using a squeezing roller. The coating layer was then cured exposing both sides of the treated fabrics to a UV lamp. Thermogravimetric (TG) measurements showed that thermal stability and char formation of the treated fabrics increases with increasing the concentration of vinyl phosphonic acid. Furthermore, LOI values improved with increasing the concentration of vinyl phosphonic acid in the adhesive formulation.

### 4.3. Triazine-Based Flame Retardants

Cyanuric chloride is the main starting material for obtaining triazine-based flame retardants: indeed, this product shows a high chemical reactivity and is suitable for synthesizing phosphorus-nitrogen flame retardants with different phosphorus to nitrogen ratios, either via nucleophilic substitution or Michaelis-Arbuzov reaction.

**Figure 10:**
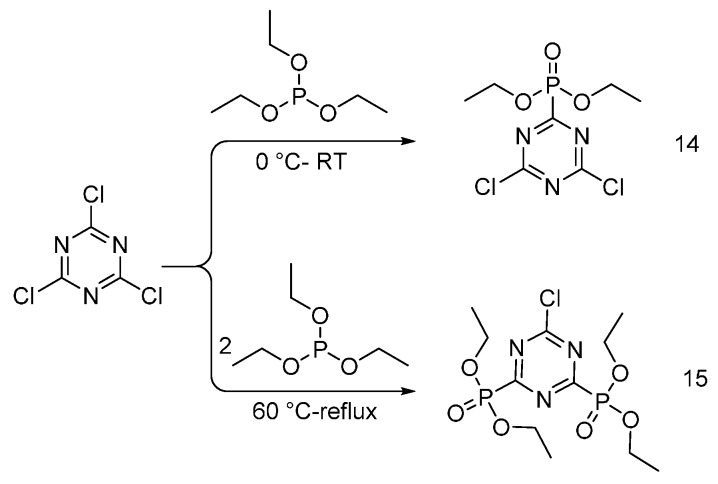
Representative synthesis of monomers (**14**) and (**15**) via Michaelis-Arbuzov reaction.

Chang and co-workers synthesized diethyl 4,6-dichloro-1,3,5-triazin-2-ylphosphonate (**14**), tetraethyl 6-chloro-1,3,5-triazin-2,4-diyldiphosphonate (**15**) via Michaelis-Arbuzov reaction (as shown in Figure 10), and used these monomers for conferring flame retardant properties to cotton fabrics [26].

The chlorine atoms of the two synthesized oligomers were exploited to react with hydroxyl group of the cellulose, through the formation of ether bonds, hence permanently linking the FR additives to the cellulosic substrate [27]. Thermogravimetric analyses showed the formation of a char at 600 °C, which proved the condensed phase mode of action of the synthesized FRs. LOI values were found to increase up to 48% (for treated fabrics) with increases in the flame retardant concentration. From an overall point of view, the synthesized oligomers acted as good flame retardants for cotton fabrics when at least 10 wt % add-on was achieved. As triazine-based flame retardants are believed to be of interest for textile industries, the durability of the flame retarded cotton fabrics has not been reported yet.

**Figure 11:**
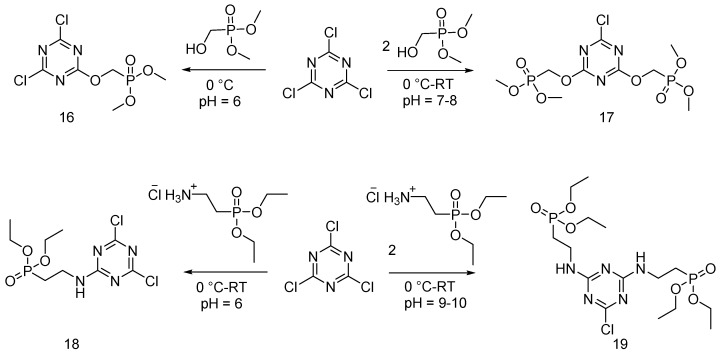
Synthesis of flame retardants (**16**)–(**19**) via nucleophilic reaction.

Slopek and co-workers exploited a nucleophilic reaction for synthesizing dimethyl (4,6-dichloro-1,3,5-triazin-2-yloxy)methyl phosphonate (**16**), (6-chloro-1,3,5-triazine-2,4-diyl)bis(oxy)bis(methylene) diphosphonate (**17**), diethyl 2-(4,6-dichloro-1,3,5-triazin-2-ylamino)ethylphosphonate (**18**) and tetraethyl 2,2’-(6-chloro-1,3,5-triazine-2,4-diyl)bis(azanediyl)bis(ethane-2,1-diyl) diphosphonate (**19**). The reactions are shown in Figure 11.

The obtained FR products were applied onto cotton fabrics [28,29]. From an overall point of view, the treated cotton fabrics showed a fire behavior similar to that provided by monomers (**14**) and (**15**).

**Figure 12:**
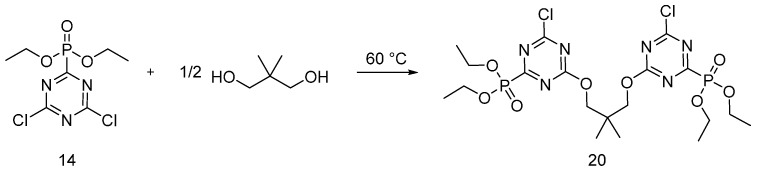
Schematic synthesis of oligomer (**20**).

Recently, dioxo(3-triethylphosphite-5-chlorin-triazine) neopentyl glycol (**20**) was synthesized from reaction of monomer (**14**) with almost half equivalent of neopentyl glycol (Figure 12); the obtained product was applied onto cotton fabrics [30].

Cotton fabrics were impregnated in a solution containing (**20**) and Na_2_PO_4_·H_2_O as a catalyst at 40 °C. After drying, the samples were then cured at 160 °C. LOI values increased with increases in the concentration of the oligomer in the treated cotton fabrics. Furthermore, the effect of the catalyst concentration on the treated fabrics was investigated: it was found that with increases in the catalyst concentration, the LOI values of the treated fabrics increased. This finding was ascribed to the presence of the catalyst that promoted the grafting reaction between the oligomer and the fabric substrate. Vertical flammability tests showed a gradual decrease of char length and increase of the char residue with increases in the FR concentration. Furthermore, with increases in the flame retardant concentration to at least 20 wt %, the treated samples did not show any afterglow phenomena. Finally, the flame retarded fabrics showed a significant washing fastness that makes the synthesized oligomer a durable flame retardant for cotton fabrics.

**Figure 13:**
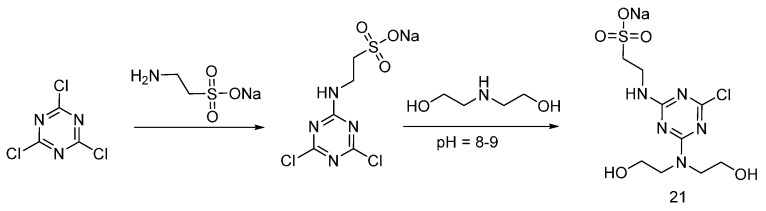
Synthesis of S-N flame retardant containing triazine (**21**).

Zhang et al. prepared a sulfur-Nitrogen flame retardant containing triazine ((**21**), Figure 13) by reacting cyanuric chloride with sodium 2-aminoethane-sulfanilate, and then with diethanolamine [31].

The obtained flame retardant was dissolved in water at different concentrations and applied onto cotton fabrics. The thermal stability of the treated fabrics was investigated using TG analyses in air atmosphere. The treated cotton fabrics showed an increase of the char residue at 600 °C, which proved the condensed phase mode action of the obtained flame retardant. However, no standard burning tests were reported and therefore it was not possible to assess the flame retardant performance of the treated cotton fabrics.

**Figure 14:**
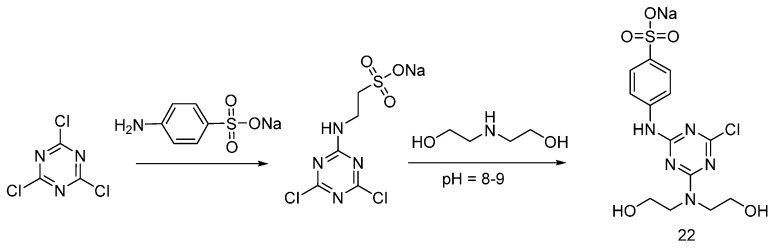
Synthesis of S-N flame retardant containing triazine (**22**).

A similar approach was adopted by Zhao and co-workers, who synthesized the flame retardant **22** (shown in Figure 14), suitable for cotton fabrics [32].

The flame retardant performances of the treated fabrics were evaluated using LOI and vertical flammability tests. Cotton fabrics could reach self-extinction when treated with 10 wt % add-on of the FR.

**Figure 15:**
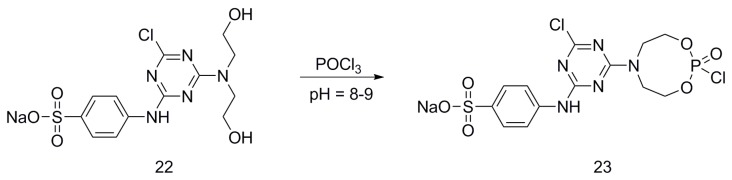
Representative synthesis of monomer (**23**).

Pursuing this research, (**22**) was reacted with POCl_3_, affording 6-chloro-4-(diethylamino phosphorate phosphoryl chloride)-2-(sodium 4-aminobenzensulfonate)-1,3,5-triazine (**23**) (Figure 15) [33].

### 4.4. Hybrid Organic-Inorganic Flame Retardants

Hybrid organic-inorganic flame retardants are materials that usually favor the formation of a carbonaceous layer upon exposure to a heat source [34]. Among the different strategies that can be successfully exploited, the sol-gel technique is one of many techniques developed for incorporation of these hybrid organic-inorganic flame retardants onto textiles [35].

**Figure 16:**
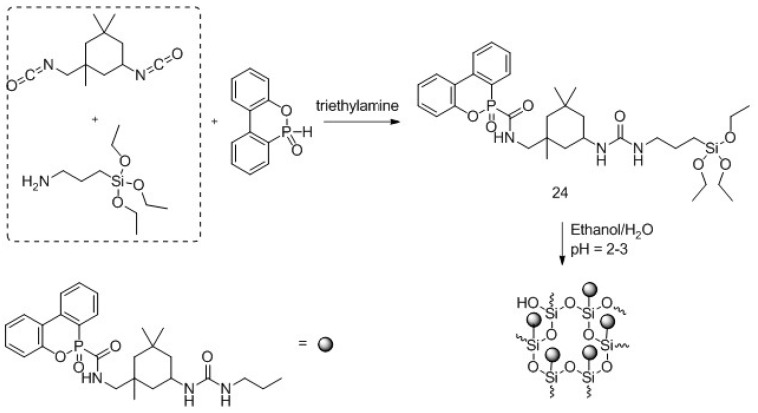
Representative synthesis of hybrid organic-inorganic flame retardant precursor (**24**) and its hydrolysis.

In this context, several phosphorus-based silicon containing flame retardants have been developed. Hu and co-workers [36] exploited the reaction of isophorone diisocyanate with (3-aminopropyl)triethoxysilane and 9,10-dihydro-9-oxa-10-phosphaphenanthrene-10-oxide (DOPO), obtaining oligomer 24 of Figure 16.

After the hydrolysis of the product in acidic media, a sol-gel solution was obtained and used for treating cotton fabrics by dipping. The treated samples were then dried in the oven at 80 °C and then 130 °C. TG analyses showed a decreased onset temperature and an increased char with increases in the concentration of the sol-gel precursor on cotton fabrics: these findings were attributed to the acid environment promoted by the degradation of the organophosphorus part and to the thermal stability of Si–O bonds that exerted a protection on the underlying fabric. A similar trend was found in MCC tests: PHRR and HRC values showed a decrease with increases in the concentration of the precursor. All these results proved that the sol-gel precursor acts in the condensed phase by increasing the char formation of the treated cotton samples, which reduces the heat release.

**Figure 17:**
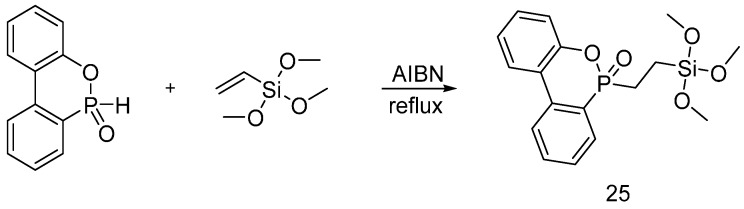
Synthesis of hybrid organic-inorganic flame retardant (**25**).

Quite recently, Vasiljević and co-workers [37] exploited an addition reaction of DOPO to vinyltrimethoxysilane in the presence of Azobisisobutyronitrile (AIBN) as initiator, giving rise to the formation of the flame retardant (**25**) shown in Figure 17.

Different concentrations of the obtained flame retardant were prepared in ethanol and were hydrolyzed using HCl. The cotton fabrics were then treated by the pad-dry-cure method at 20 °C, subsequently dried at 120 °C and then cured at 150 °C. TG analyses showed a decrease of the thermal stability and an increase of the char formation with increases in the amount of dry solid on the treated cotton fabrics. In addition, the flame retardant performance of treated cotton fabrics was investigated using vertical flame spread tests: by increasing the solid dry add-on, an increased flaming combustion after the removal of the flame was found; conversely, LOI values were improved.

Pursuing this research, the same group exploited the combination of the sol-gel precursor (**25**) and tetraethoxysilane (TEOS), which was utilized as a sol-gel finishing for polyamide 6 (PA6) fabrics [38]. The obtained hybrid system decreased the Total Heat Release values of the treated fabrics, increasing, at the same time, the char yield with respect to the use of precursor (**25**) alone, hence proving the synergistic behavior of the combined hybrid system.

**Figure 18:**
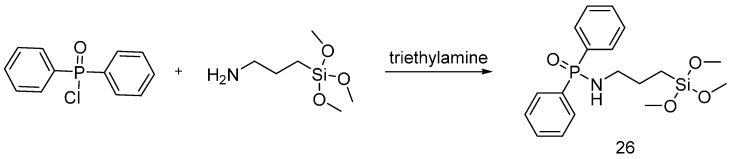
Synthesis of hybrid organic-inorganic flame retardant (**26**).

Yang et al. [39] reacted (3-aminopropyl)trimethoxysilane with diphenylphosphinic acid in order to prepare the flame retardant precursor (**26**) shown in Figure 18.

The obtained precursor was dissolved in aqueous/ethanol solutions at different concentrations and applied as a hybrid organic-inorganic flame retardant for cotton fabrics. After treatment, the fabrics were dried at 140 °C. The thermal stability and the flammability behavior of the treated fabrics were assessed; furthermore, the washing fastness was evaluated. TG measurements showed an increase of char formation of the treated cotton fabrics at 500 °C. Vertical flame spread tests revealed char formation quickly after ignition and the specimens achieved self-extinction after the removal of the flame. In addition, the treated fabrics did not lose their flame retardancy after several laundry cycles, thus proving the durability of the proposed flame retardant treatment.

**Figure 19:**
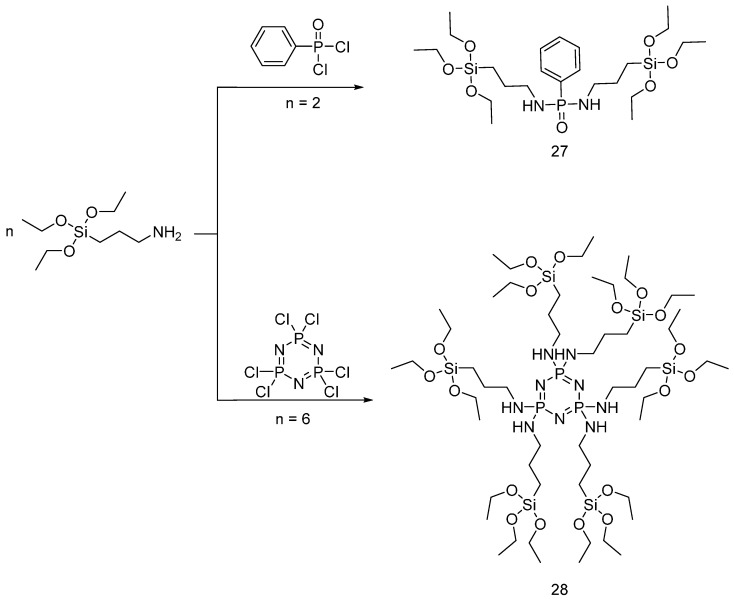
Representative schematic synthesis of precursors (**27**) and (**28**).

Very recently, Liu et al. exploited a similar synthetic approach for obtaining hybrid organic-inorganic 3-aminopropyl triethoxysilane derivatives on cotton fabrics [40]. To this aim, a nucleophilic substitution was carried out (Figure 19). A 10 wt % solution of the hybrid precursor (**27**) was prepared and then the cotton fabrics were immersed for different times before a subsequent thermal treatment.

In a similar way, Wang and co-workers synthesized the precursor (**27**) shown in Figure 19, which was applied onto cotton fabrics at different concentrations [41]. TG data of the fabrics treated with precursor (**27**) showed an increase of the thermal stability with increases of final dry add-on. Conversely, the cotton fabrics treated with precursor (**28**) exhibited a lower thermal stability as compared to the other hybrid system. However, both flame retardants showed an increase of the char residue with increases in the add-on on the treated fabrics. A similar trend was found when the treated fabrics were subjected to vertical flammability tests. Therefore, from an overall point of view, the designed hybrid coating systems were able to provide improved fire retardancy to cotton, exploiting the formation of a thermally stable char that prevents the underlying substrate from ignition and limits the release of flammable gases during the thermal degradation process. Finally, the washing fastness of the flame retardant was assessed: in particular, the specimens treated with the sol-gel precursor (**28**) did not exhibit any change of the phosphorus content after the first washing cycle.

### 4.5. Flame Retardants as Polymer-Additives

Another possible strategy, differing from those described in the previous paragraphs, exploits the design of phosphorus-based polymeric flame retardants, rather than low molecular weight counterparts. Indeed, the former are being widely investigated because of their durability and ecofriendly character: this has pushed the scientific community to employ different polymeric flame retardants as additives for either textiles or engineering plastics. In addition, polymeric flame retardants bearing hydroxyl or carboxyl groups were found to be very effective for cellulosic materials.

**Figure 20:**
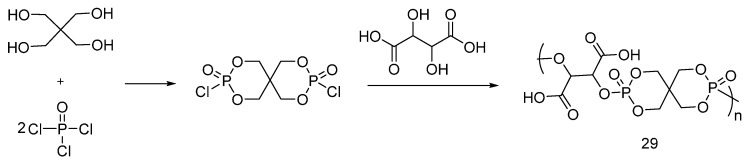
Synthetic approach of poly(1,2-dicarboxyl ethylene spirocyclic pentaerythritol bisphosphonate).

Liu and co-workers [42] synthesized poly(1,2-dicarboxyl ethylene spirocyclic pentaerythritol bisphosphonate) (**29**) by reacting spirocyclic pentaerythritol bisphosphorate disphosphoryl chloride with tartaric acid (Figure 20). Cotton fabrics were washed with NaOH solution and then soaked in an aqueous solution containing 30 wt % of (**28**) and a catalyst. After drying, the samples were cured at 160 °C and washed with sodium carbonate solution and then dried at 70 °C.

The flame retardant performance was investigated by LOI, vertical burning tests and TG analyses. LOI values showed an increase up to 33.8% by increasing the add-on up to 21.2 wt %. Furthermore, vertical burning tests performed on the treated samples showed a reduction of the afterglow time and of the char length, without after-flame phenomena, as compared to the untreated fabric. As assessed by TG analyses, an anticipation of the onset thermal decomposition, as well as an increase of the char formation by increasing the add-on was found: once again, this latter finding proves the condensed phase mode of action of the flame retardant.

**Figure 21:**
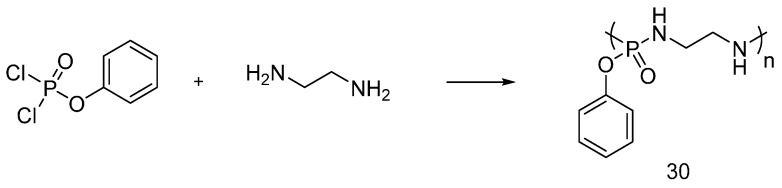
Representative synthesis of poly(phoshorodiamidate) (**30**).

Dong et al. reacted phenyl dichlorophosphate with ethylenediamine, in order to obtain the poly(phoshorodiamidate) (**30**), shown in Figure 21. The product was applied onto cotton fabrics [43].

More specifically, cotton fabrics were dipped in solutions of (**30**) at different concentrations, in the presence of tetraethyl orthosilicate employed as a crosslinker to permanently link the polymer to the fabric substrate. The samples were then dried and cured at 160 °C. All samples were then washed before testing. The flame retardant performance of the treated cotton was investigated through vertical burning tests: as compared with untreated fabrics, shorter after-glow time and char lengths were found by increasing the add-on of the treated samples.

**Figure 22:**
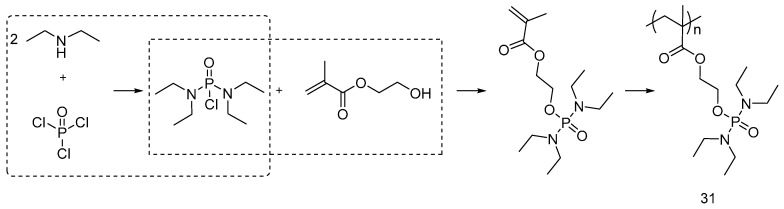
Representative synthesis of methacryloyloxyethylorthophosphorotetraethyl-diamidate polymer.

Abou-Okeil et al. [44] exploited the bulk polymerization process of methacryloyloxyethylorthophosphortetraethyl-diamidate monomer in the presence of benzoyl peroxide as radical initiator, for synthesizing polymer (**31**) exploiting a three-step reaction (Figure 22).

Cotton fabrics were dipped into aqueous solutions of polymer (**31**) at different concentrations, in the presence of a binder and a crosslinker. The treated fabrics were then squeezed, dried and then cured at 150 °C. After the treatments, the fabrics increased their LOI values; TG measurements revealed the formation of a stable char. Despite these results, the observed changes were not very significant as compared to untreated cotton.

**Figure 23:**
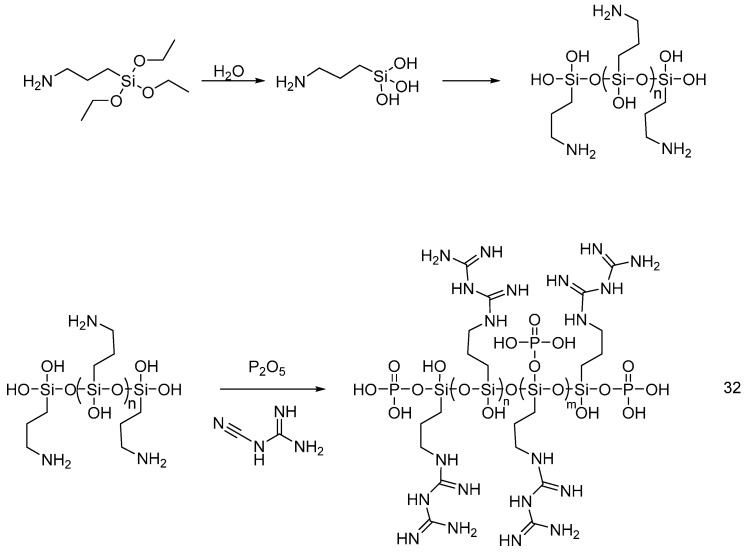
Schematic synthesis of guanidyl- and phosphorus-containing polysiloxane (**32**).

Recently, Dong et al. [45] synthesized a guanidyl- and phosphorus-containing polysiloxane flame retardant (**32**): for this purpose, according to Figure 23, 3-aminopropylethoxysilane was hydrolyzed and then reacted with dicyandiamide and P_2_O_5_ (Figure 23).

After washing, cotton fabrics were soaked in a bath containing the FR polymer, 2-phosphonobutane-1,2,4-tricarboxylic acid and urea at room temperature, then squeezed in laboratory scale padder, rinsed with water, dried at 80 °C and cured at 160 °C. The flame retardant performance of the treated samples was tested by cone calorimetry and thermogravimetric analyses. Cone calorimetry measurements showed that by increasing the add-on of the FR polymer, HRR and THR decreased accordingly, hence, indicating that the flame retardant hinders the formation of combustible volatile species. In addition, the CO_2_/CO ratio decreased dramatically with increases in the FR add-on. TG measurements performed on the treated cotton fabrics showed an increase of the decomposition temperature with, at the same time, an increase of char formation. It is worthy to mention that, in addition to the enhanced flame retardant performances, the treated fabrics showed an effective antimicrobial behavior against *E. coli* and *S. aureus* [46].

**Figure 24:**
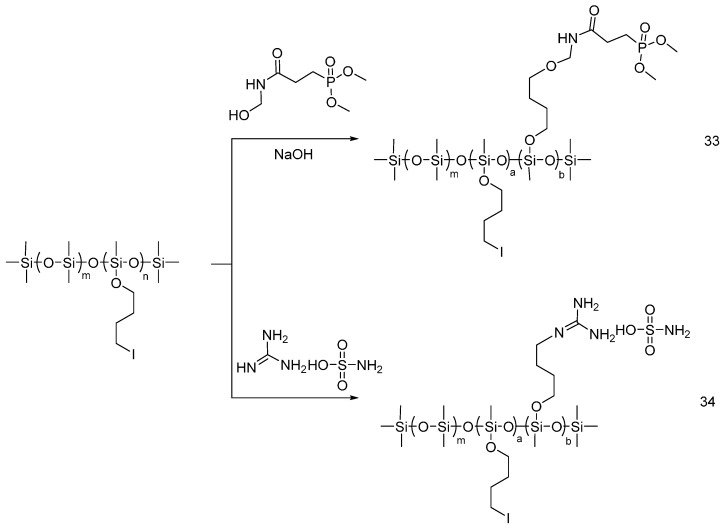
Representative synthetic approach for flame retardants (**33**) and (**34**).

Very recently, the same group synthesized a novel nitrogen and phosphorus-containing polysiloxane ((**33**), Figure 24), starting from poly(4-iodo-n-butoxy)methylsiloxane and N-methylo-3-(dimethoxy dibenzyloxyphosphoryl) acrylic amide [47].

By adopting a similar approach, the polymeric additive ((**34**), Figure 24) was also synthesized using poly (4-iodo-n-butoxy) methylsiloxane and guanidine sulfamate [48].

Both polymeric flame retardant additives were applied onto cotton fabrics, using an impregnation bath also containing urea and zirconium oxide. The samples were then dried and cured at 160 and 150 °C (for treated fabrics with (**33**) and (**34**), respectively). Cone calorimetry and TG analyses were exploited for assessing the performances of the samples treated with polymer (**33**): more specifically, the treated fabrics showed an enhancement of the flame retardancy by decreasing HRR, THR and CO_2_/CO ratio. TG data showed a decrease of the onset decomposition temperature and an increase of the char yield for all treated samples. Furthermore, LOI values of the treated samples did not show a significant drop after several washing cycles, hence, proving the durability of flame retardant (**33**). A similar FR performance was also assessed for the cotton fabrics treated with polymer (**34**): unlike (**32**), the treated fabrics also showed water repellency, which was attributed to the reaction of the flame retardant with cotton fibers, leading to methyl group orientation on the fiber surface.

**Figure 25:**
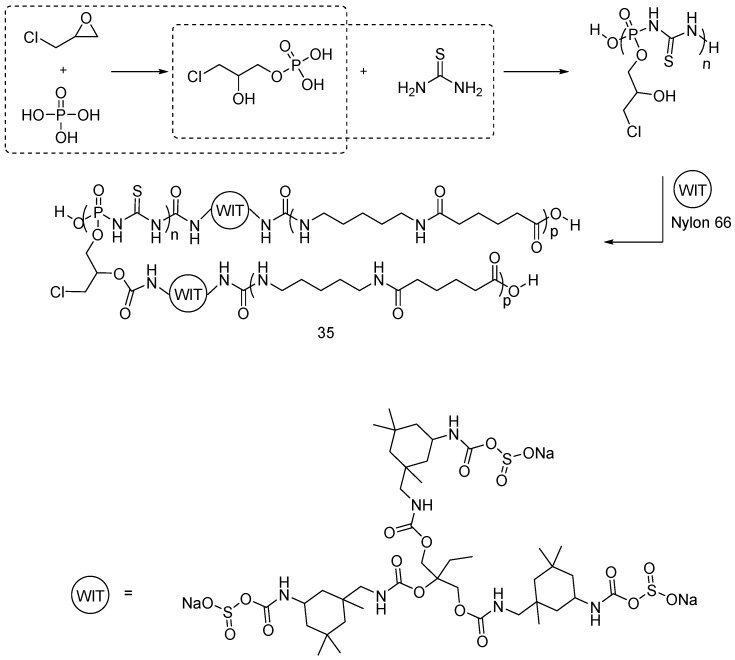
Synthetic route of polymeric additive (**35**).

Very recently, Chen and co-workers synthesized a polymeric sulfur-containing flame retardant ((**35**), Figure 25) for nylon fabrics, using thiourea, phosphoric acid, water soluble isocyanate-terminated crosslinker (WIT) and epichlorohydrin [49].

In particular, nylon fabrics were dip-treated in a finishing solution containing the FR polymer, a crosslinker and potassium thiocyanate. After drying, the samples were cured at 150 °C and then soaked in water/soap solution, rinsed with water and dried again. As assessed by TG analyses, the onset decomposition temperature of the treated fabrics was anticipated, notwithstanding a substantial increase of the char formation with respect to the untreated counterparts. MCC analyses showed an important decrease of HRR, THR and HRC, together with a significant char formation. The durability of the flame retardant treatment was also assessed: 10 washing cycles did not have any detrimental effect on the flame retardants’ performances.

### 4.6. Phosphoramidate Flame Retardants

Phosphoramidate derivatives are promising flame retardant additives, due to the synergistic effect provided by phosphorus and nitrogen.

**Figure 26:**
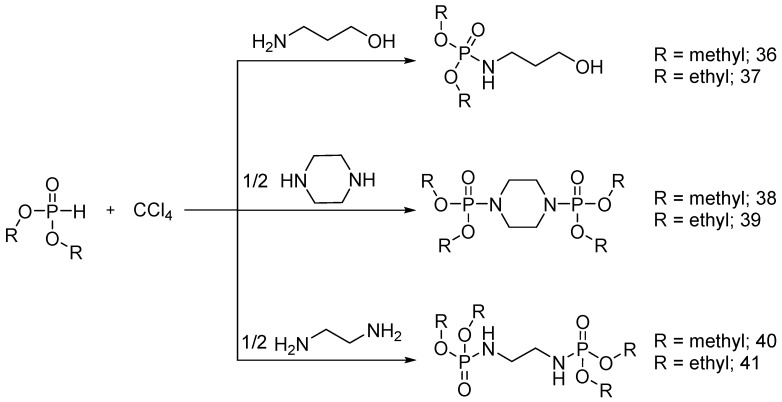
Scheme of the synthesis of phosphoramidate derivatives (**36**)–(**41**).

Gaan and co-workers [50] synthesized different phosphoramidate derivatives as flame retardant additives for cotton fabrics, following the Atherton-Todd reaction in the presence of dialkylphosphites as starting materials (Figure 26).

The products were applied onto cotton fabrics from acetone solutions with different concentrations of phosphoramidates (**36**)–(**41**). LOI values were found to increase by increasing the phosphorus content of the treated cotton.

Nguyen and co-workers further investigated the flame retardant performance of (**36**) and (**37**) at different add-ons on cotton fabrics [51]: in particular, the fabrics treated with an add-on beyond 5 wt % showed no afterflame or afterglow time, providing the fabrics with self-extinction. The effect of chemical structure on the performance of these flame retardants was studied using MCC. It was found that THR values decreased with increases in the add-on of product (**36**). Conversely, THR values were found to increase by increasing the add-on of (**37**). Product (**39**) promoted char formation and decreased the THR of the treated samples with increases in the add-on on the treated fabrics. On the other hand, vertical flammability tests performed on cotton fabrics treated with (**39**) showed an increase after flame and no afterglow phenomena [52].

**Figure 27:**
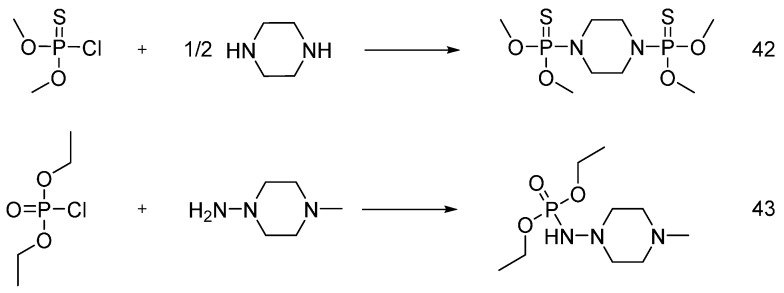
Synthesis of (**42**) and (**43**).

As a comparison to (**39**), the same group [53] synthesized new flame retardants using dimethyl phosphorochloridothionate and diethyl phosphorochloridate as starting material (Figure 27). From an overall point of view, it was found that the flame retardant performance of the fabrics treated with **39** was much better than that of the substrates treated with same add-on of (**42**). Furthermore, when cotton fabrics were treated with (**39**) and (**43**), they showed almost the same FR performances.

The phosphoramidate (**43**) was also investigated as flame retardant additives for cotton twill and cotton print cloth [55]. The flame retardant performance of the treated fabrics was found to depend on the type of treated fabrics: in particular, the add-on being equal, the treated cotton twill showed better FR performances as compared with the treated print cloth.

**Figure 28:**
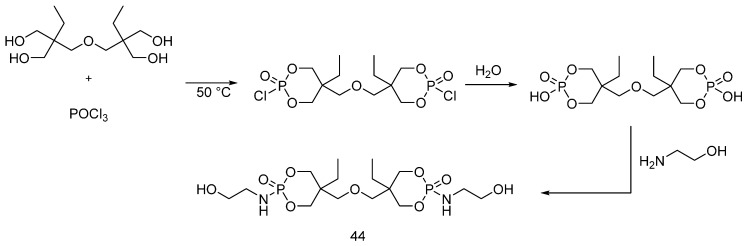
Representative schematic synthesis of (**44**).

Recently [56], ditrimethylolpropane di-N-hydroxyethyl phosphoramide (**44**) was synthesized using ditrimethylolpropane and phosphoryl chloride as starting materials (Figure 28).

The obtained FR additive (**44**) was applied to cotton, nylon and polyester fabrics, using a dipping method. The fabrics were then dried and cured at 160 °C. The flame retardant performance of the treated fabrics was tested using vertical flammability tests: it was found that the treated nylon fabrics showed better flame retardant performances as compared with the other treated fabrics.

**Figure 29:**
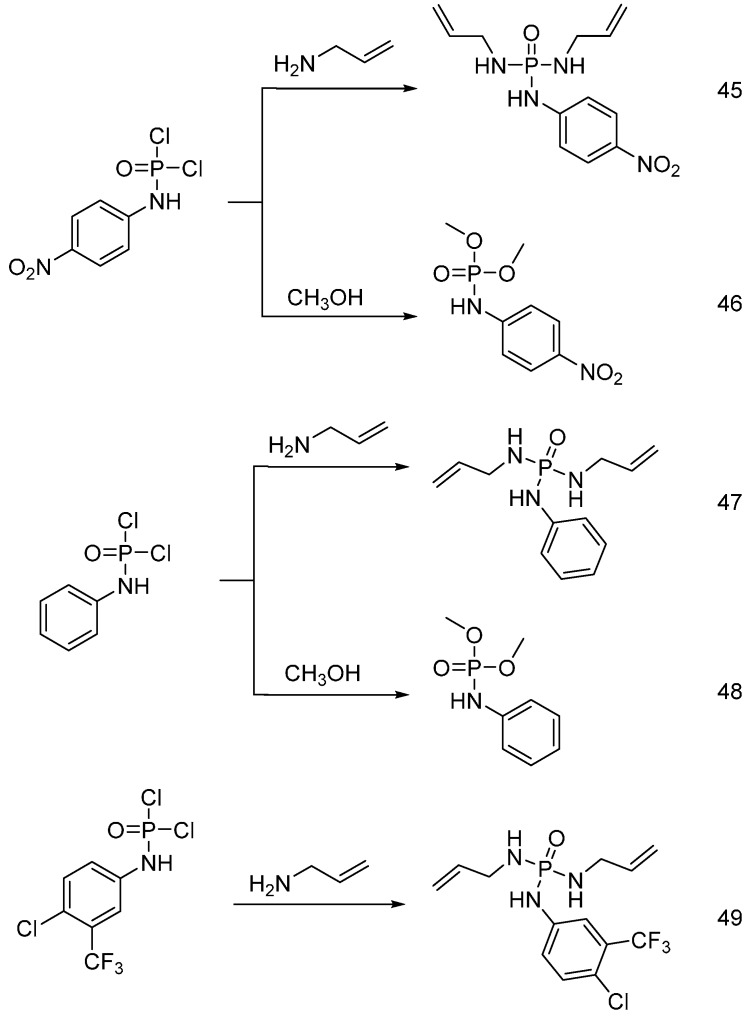
Representative synthesis of flame retardant additives (**45**)–(**49**).

Shariatinia and co-workers [57] synthesized different phosphoramidates and phosphoramides starting from their corresponding phosphoramidic dichloride in an ultrasonic bath, obtaining the flame retardant additives (**45**)–(**49**) shown in Figure 29. All these flame retardant additives were applied to cotton fabrics. The cotton fabrics were treated with an aqueous solution of each flame retardant additive at different concentrations and in the presence of a thermal initiator. The treated samples were then dried and cured at 121 °C and their flame retardant performance was assessed through vertical burning tests: the flame retardant additives (**46**) and (**48**) showed a lower degree of grafting and an increased char length, as compared with the samples treated with the same concentration of (**45**), (**47**) and (**49**) additives. In general, the char length was found to decrease with increases in the degree of grafting, which in turn depended on the additive concentration in the corresponding treatment solutions.

### 4.7. Miscellaneous and Potential Phosphorus-Based Flame Retardants for Textile Applications

In addition to the list of chemical structures and chemical classifications that are mentioned earlier, it is worth listing some miscellaneous and promising flame retardants based on their chemical structure and behavior (Table 2).

## 5. Phosphorus-Containing Biomacromolecules as Flame Retardants

The design of environmentally friendly flame retardant systems for textiles involves another possible strategy, recently studied [66,67,68], which is based on the use of some biomacromolecules (in particular proteins like caseins, hydrophobins and whey proteins, and DNA—deoxyribonucleic acid). Indeed, the chemical structure of some of these products shows the presence of phosphorus and other elements (like nitrogen and/or sulphur), which can confer flame retardant features to different fibers and fabrics.

The use of biomacromolecules as FRs represents a potential innovative strategy that goes far beyond the chemical approach behind the design of standard flame retardants for textiles. Up to 5 years ago, the utilization of these biomacromolecules was undoubtedly delineated to other application fields. Among them, biomacromolecules have been used as edible films, adhesives, food emulsifiers, printing, papermaking, leather finishing systems, as well as for the design of biosensors and environmental monitoring systems.

Several advantages can be conferred from the exploitation of proteins and DNA in providing FR features to textiles [67]: in particular, their ease of manipulation, the possibility of exploiting application techniques that are already designed and optimized for fabric finishing (like impregnation/exhaustion, spray, or even the layer-by-layer deposition [69]) and the set-up of low impact/sustainable finishing recipes (thanks to the use of water-based solutions/dispersions).

In addition, caseins and whey proteins are somehow by-products or even wastes recovered from the agro-food industry: therefore, their use as possible FRs may represent a new way toward the valorization of agro-food crops, reducing and/or preventing their landfill confinement. Finally, the high price of DNA with respect to standard chemical FRs is being overcome, as large scale extraction and purification processes have been developed [70].

The next paragraphs will describe the main outcomes and accomplishments resulting from the use of these P-based sustainable products as a valuable, potentially eco-friendly alternative to traditional chemical FRs for cotton, polyester and cotton-polyester blends. Table 3 summarizes the main results achieved so far. In particular, some fresh examples of textile fireproofing provided by these phosphorus-based biomacromolecules will be thoroughly described, highlighting some structure–fire behavior relationships for the chosen biomacromolecules.

### 5.1. Caseins

Caseins are phosphorylated proteins that represent the main fraction of milk proteins (around 80%) and, possibly, the most widely investigated food proteins, obtained as co-products during the production of skim milk.

Their main constituents are:

- α_S1_-caseins: They contain eight or nine bound phosphate groups/mol and represent the major protein fraction of bovine milk;
- α_S2_-caseins: They show a structure similar to α_S1_-casein, as they contains, according to its four differentially phosphorylated isoforms, around 10–13 phosphate groups/mol;
- β-caseins: They are less phosphorylated with respect to α_S1_- and α_S2_- counterparts. They include glutamines bearing amino groups and they display a single major phosphorylation site near the N-terminus. Bovine β-caseins can be sourced in a single, fully phosphorylated form containing five phosphate groups/mol;
- κ-caseins: As regard to any other casein, these constituents have the smallest phosphate component. In particular, the phosphorylated regions, present as single sites, are positioned in the C-terminal region of the biomacromolecule.

Notwithstanding their standard cheese farming uses, these proteins have been mainly employed as a food ingredient for improving different physical properties like foaming and whipping, water binding and thickening, texture and emulsification. Furthermore, they have been exploited as coatings, specifically referring to papermaking, leather finishing, printing and manufacturing of synthetic fibers [77].

As far as fire retardancy goes, cotton, polyester and cotton polyester blend fabrics (polyester content: 65 wt %) were treated with a caseins aqueous suspension (5 wt %) in a climatic chamber (30 °C and 30% R.H.): the suspension was spread onto the samples with a spatula and the excess was removed by gently pressing with a rotary drum; then the coated samples were dried to constant weight. The final dry add-on was 20 wt % [71,72].

The presence of the casein coatings promoted a strong anticipation of both cellulose and polyester decomposition: this behavior, observed for all the types of treated fabrics, was attributed to the phosphate groups located on the shell of casein micelles, which, upon heating, release phosphoric acid that favors the degradation of cellulose or polyester toward the formation of a stable char. This latter exerts a protective effect on the underlying textile, limiting the oxygen diffusion and absorbing the heat evolved during the combustion [78].

Therefore, the anticipation of the fabric degradation occurs, but, at the same time, is accompanied by the formation of a thermally stable char.

The fire behavior of the treated fabrics was assessed by flammability tests performed in horizontal configuration and by LOI tests. In the presence of the casein coatings, a remarkable reduction of the total burning rate and a strong increase of the final residue were observed, irrespective of the type of fabric. In particular, the casein coatings provided self-extinction to cotton fabrics, even after a second flame application; as far as PET is considered, the protein coatings significantly reduced its burning rate and blocked the flame propagation within 30 mm, giving rise, at the same time, to a remarkable increase of the final residue. Conversely, the biomacromolecule was not able to suppress the melt dripping of the synthetic fabric. Furthermore, the protein coatings were responsible for a notable increase of LOI values of both cotton and polyester (respectively +6% and +5% as compared to the untreated fabrics); on the other hand, they did not considerably modify the LOI value of the blend.

Finally, cone calorimetry tests were performed at 35 kW/m^2^ heat flux: both time to ignition and combustion rate were strongly influenced by the presence of the casein coating, irrespective of the type of fabric substrate. In particular, despite a significant TTI decrease (indeed, the fabrics were found to ignite earlier in the presence of the proteins), the peak of heat release rate showed a substantial decrease for coated cotton (−19%) and for the blend (−15%). Finally, the char-forming character of the proteins on PET was confirmed by a significant increase of the final residue.

### 5.2. Deoxyribonucleic Acid

It is very well known that deoxyribonucleic acid (DNA) consists of two long polymer chains of nitrogen-containing bases—namely, adenine (A), guanine (G), cytosine (C) and thymine (T)—with backbones made of five-carbon sugars (i.e., the deoxyribose units), as well as of phosphate groups tied by ester bonds. The chains are rolled-up around the same axis and bonded together: this way, a double helix is formed, which exploits the presence of H-bonds between the bases located side by side and paired in a specific mode (cytosine bases are combined with guanine, while adenine bases are paired with thymine).

In the obtained 3D organization, the phosphoric residues and deoxyribose units are oriented toward the outside of the biomacromolecule; conversely, the paired bases are located in the inner portion of the polymer and are stabilized by hydrophobic interactions.

The ability of DNA to form double-stranded arrangements has been already utilized for obtaining several DNA-based nanomaterials, exploitable for applications ranging from life science to computing: some examples refer to DNA-linked metal nanoparticles, DNA-directed nanowires and DNA-functionalized carbon nanotubes [79,80].

The use of DNA as a sustainable flame retardant is quite recent and can usually be attributed to the structure of this biomacromolecule, which represents an all-in-one intumescent system [73,74,75,81,82,83]. More specifically, the double helix comprises all three constituents of an intumescent material, namely: (i) the phosphate groups that form phosphoric acid; (ii) the deoxyribose units, which act as a carbon source and as blowing agents and (iii) the four nitrogen-containing bases that may give rise to the formation of NH_3_.

Like an intumescent material, when DNA is exposed to a heat source, it develops a multicellular foamed char, which limits the heat and mass transfer between flame and polymer, hence providing flame extinction.

The first pioneering work dealing with the flame retardant properties of this biomacromolecule was published in 2013 and dealt with the use of DNA derived from herring sperm for self-extinguishing cotton fabrics. To this aim, a standard impregnation/exhaustion method for reaching the desired final dry add-on (19 wt %) was employed [74]. Among the most important achievements, an improved thermal and thermo-oxidative stability of the treated fabrics in nitrogen and air, in terms of char residue formed at high temperatures, was demonstrated. Furthermore, the combustion was blocked, reaching the flame out within 2 s, after the application of a methane flame in horizontal configuration for 3 s to the fabrics treated with this biomacromolecule. The remarkable flame retardant features of DNA were further confirmed by LOI (28 vs. 18% for DNA-treated fabrics and untreated cotton, respectively) and cone calorimetry tests: these latter showed that none of the DNA-treated specimens ignited upon exposure to 35 kW/m^2^ heat flux.

The high char-forming character of deoxyribonucleic acid was attributed to its chemical structure, where the phosphate groups are able to form phosphoric acid that catalyzes the dehydration of cotton, favoring its auto-crosslinking toward the formation of a stable aromatic char (that is also formed by the deoxyribose units) and inhibiting the production of volatile flammable species. This char behaves as a physical protective barrier on the underlying substrate, limiting the heat, fuel and oxygen transfer between the fabric and the flame. At the same time, the decomposition of pyrimidine and purine bases could promote the formation of azo-compounds able to further induce the char development and the formation of non-combustible gases, like nitrogen, carbon monoxide and carbon dioxide.

Pursuing this research, the effect of different DNA add-ons on cotton flammability was investigated: for this purpose, three different add-ons (namely 5, 10 and 19 wt %) were deposited on cotton fabrics, exploiting the same impregnation/exhaustion method [75]. Similarly to other biomacromolecules, the presence of the coating strongly anticipated the cellulose decomposition, which was directly related to the DNA add-on: in particular, as assessed by TG analyses in nitrogen and air, the higher was the biomacromolecule add-on, the lower was the temperature, at which degradation started to occur. This finding was attributed to the phosphate groups of the biomacromolecule, which decompose at about 200 °C, releasing phosphoric acid, hence favoring the dehydration of the fabrics, and finally giving rise to the formation of a residue, thermally stable up to 500–600 °C [73,83]. Flammability tests performed in horizontal configuration clearly demonstrated that the self-extinction of the treated fabrics strictly depends on the biomacromolecule add-on: in particular, the fabrics having the lowest DNA add-on (i.e., 5 wt %) burnt completely, while the other specimens (having 10 or 19 wt % add-on) were able to achieve the flame out. SEM-EDS measurements of the char zone of the residue left by the self-extinguishing coatings proved that the original texture of the fabrics was still preserved and the fibers appeared almost undamaged. Furthermore, their surface was covered by small spherical structures finely dispersed, mainly consisting of C, O and P elements: this was a clear indication of the intumescent character of the biomacromolecule.

Pursuing this research, very recently, Bosco et al. deeply studied the effect of different parameters (i.e., molecular size, pH of aqueous solutions, number of impregnations) on the fire behavior of deoxyribonucleic acid [75]. Flame spread and cone calorimetry tests showed that the coatings derived from low molecular size DNA significantly improved the fire behavior of the fabric substrate, which they were applied to. In addition, the fire behavior was better enhanced when multiple impregnations were used instead of a single one, for achieving the same final add-on. In particular, the use of multiple impregnations at pH 4 and 8 provided 86% and 74% of the tested samples with self-extinction, respectively; furthermore, these treated fabrics showed 45% and 25% reduction of THR and pkHRR when were exposed to 35 kW/m^2^ heat flux, with respect to untreated cotton. These results were attributed to the higher ease of diffusion of the low molecular size DNA coating within the fabric microfibrillar surfaces, and to its higher thermal stability in air.

Quite recently, DNA was also exploited as a component of layer by layer (LbL) coatings: indeed, its assemblies were found to improve the flame retardant features of the treated fabrics, keeping, at the same time, a green character.

**Figure 30:**
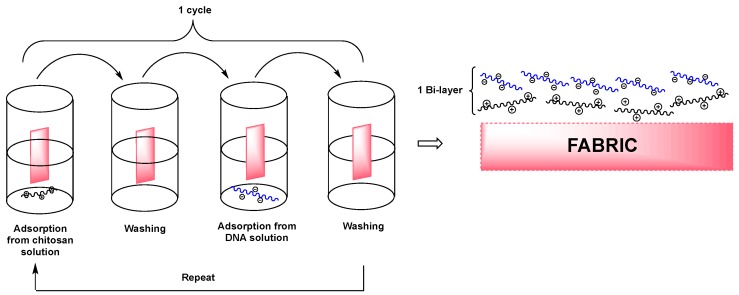
Scheme of the chitosan-DNA LbL assembly.

In particular, Carosio and co-workers [76] combined DNA (negatively charged) with chitosan layers (positively charged) on cotton, thus building up an assembly containing up to 20 bi-layers. The structure of the obtained LbL assembly is schematized in Figure 30.

The formation of the LbL architecture was investigated and confirmed through infrared spectroscopy and SEM analyses. In the presence of chitosan, DNA layers were found to favor the char forming character of the former biomacromolecule. In particular, the deposited coatings were able to provide cotton fabrics with self-extinction in horizontal flame spread tests and to increase LOI values from 18% (uncoated cotton) to 24% (cotton treated with 20 bilayers). Finally, cone calorimetry tests showed a HRR decrease of 40% and a remarkable increase of the final residue for the fabrics treated with the highest number of layers.

## 6. Conclusions

This work has clearly depicted the wide potentialities offered by the use of novel phosphorus-based flame retardants as a real (and not only potential) alternative to the toxic flame retardant products. Indeed, the wide possibility of tailoring the structures of the phosphorus-based additives allows exploiting an almost infinite number of FR systems that show high efficiency and suitability for different fibers and fabrics. Furthermore, the concurrent presence of phosphorus and other elements (like nitrogen, silicon and sulphur) may be very important in order to develop additive or synergistic effects during the exposure of the treated textiles to a flame or to a heat source.

Though the phosphorus based flame retardants are being developed as an alternative to toxic halogenated counterparts, before they can find real application, they need to be screened thoroughly for toxicity. It is encouraging to see that there are some efforts by researchers where the toxicity of new phosphorus based flame retardants has been evaluated and these latter have shown a low toxicity profile [7,8].

Despite the high flame retardant efficiency observed for most of the chemical and low impact FRs, there are still some unresolved issues that deserve further attention.

First of all, the design of any new P-based flame retardant product requires large investment either for assessing its suitability for the desired application (especially from a toxicological point of view) or for scaling-up the production from a lab-scale quantity to at least a pre-industrial scale, trying to maximize yields and reduce expenses.

Particularly referring to biomacromolecules, the possibility of adjusting the green technological approach to a larger scale than the lab scale (at least to pre-industrialization) is still under assessment and the final decision will be largely based on the cost-effectiveness of the described biomacromolecules. Definitely, the cost of some of the discussed biomacromolecules, such as DNA, is presently very high: therefore, their use as low impact FRs could be foreseen only on the basis of a significant cost reduction.

Second, the industrial world working in the field of flame retardant finishing for textiles is fully conscious that any novel flame retardant product should exhibit, apart from a high efficiency, durability to laundering: At present, some of the proposed P-based FRs (like the proposed phosphorus-containing biomacromolecules) cannot accomplish this goal. Indeed, they show no washing fastness at all, or, in some cases, a limited durability up to few washing cycles. This surely represents a current drawback that limits the use of the treated textiles to those restricted applications for which durability to washing treatments is not required. Thus, further research will also have to consider the design of new strategies able to overcome this challenging issue.

## Figures and Tables

**Table 1:** Main results achieved by treating fabrics with phosphorus-based flame retardants.

Type of P-based FR	Textile material	Highlights	Durability (washing fastness)	Ref.
Dioxaphosphorinane derivatives	PET	New oligomers were synthesized and their burning behavior was compared to Antiblaze 19^®^. The new oligomers showed higher thermal stability and more char residue comparing to Antiblaze 19^®^. The thermal stability and flame retardant properties were studied only on the oligomeric derivatives. Treated PETs with these oligomers were not investigated.	ND *	[20]
UV-curable flame retardants	Cotton	Cotton fabric was treated with UV-curable flame retardants and cured under UV-lamp in presence of photoinitiator. LOI values increased with increasing the FR content. MCC showed a decrease in HRC and HRR and THC with increasing the FR content. As increasing the monomer concentration and UV exposure time, the coating yield increased. Relatively small monomers were not suitable for UV-curing as they might evaporate during curing process.	Yes	[21,22,23]
	Cotton and Cotton/polyester blend	Allyl-functionalized polyphosphazene additive was investigated, avoiding the disadvantage of small molecules. The treated fabrics showed good flame retardancy with char formation. The additive acts in the condensed phase mode of action	Yes	[24]
	Polyester/Polyamide blend	UV-curable epoxy based oligomer formulation. Vinyl phosphonic acid (VPA) was incorporated in the formula. The thermal stability, char formation and LOI values of the treated fabrics increased with increasing the concentration of the VPA acid The peel strength values increased gradually up to 50.8 N/cm with increasing VPA.	ND	[25]
Triazine-based flame retardants	Cotton	The triazine-based flame retardants are derivatives of cyanuric chloride. These additives are considered as active flame retardants, forming ether bond by replacement the chlorine atoms with the hydroxyl groups pf cellulose. The fabrics were treated with flame retardants by impregnation. Thermal analysis showed a char formation which proves the condensed phase mode of action. LOI values increased with increasing the add-on of flame retardants. With increasing the flame retardant concentration, the treated fabrics did not show any afterglow, which can be considered as self-extinction.	Yes	[26,27,28,29,30,31,32,33]
Hybrid organic-inorganic flame retardants	Cotton	The fabrics were treated with flame retardants using the sol-gel technique. Increasing the sol-gel precursors on treated fabrics, showed a decrease of the HRC, PHRR and TGA onset and an increase of the char residue. Increasing the solid dry add-on increased relatively the after flame with no afterglow. Increasing the solid dry add-on increased the LOI value and decrease of the THR and HRC.	Yes	[34,35,36,37,38,39,40,41]
	PA6	The PA6 samples were treated with different concentrations of equimolar mixtures of the flame retardant and TEOS. Combination of P- and Si-based additives improved the thermos-oxidative stability and combustion behavior by increasing the char residue and reducing the HRC and PHRR of treated samples, respectively.	ND	[38]
Polymeric flame retardant additives	Cotton	The cotton fabrics were treated by dipping/soaking in a solution of the polymeric flame retardants. Binders or crosslinkers were used when needed to bind the polymer permanently onto fabrics. LOI values of the treated fabrics showed an increase with increasing the add-on. The vertical burning test of the treated fabrics showed a reduction of the afterglow time and char length. Cone calorimetry showed a decrease of HRR THR and CO_2_/CO ratio with increasing the add-on.	Partially studied	[42,43,44,45,46,47,48,49]
	Nylon fabrics	The nylon fabrics were dip-treated in a solution containing FR and crosslinker. MCC analyses showed a decrease of HRR, THR and HRC.	Yes	[49]
Phosphoramidate derivatives	Cotton	LOI values of the treated fabrics increased with increasing the phosphorus content. The treated fabrics with add-on beyond 5 wt % were found self-extinction. The thermal stability and the flame retardancy performance of phosphoramidates are influenced by the nature of chemical substituents and type of cotton fabrics.	Partially studied	[50,51,52,53,54,55,56,57]
	Nylon and polyester	The vertical flame test showed better flame retardancy of treated nylon fibers.	ND	[56]

* Not determined.

**Table 2:** Miscellaneous and potential promising flame retardants for textile applications.

Chemical structure	Textile material	Highlights	Ref.
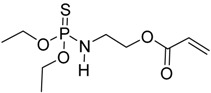	cotton	The FR monomer was grafted onto cotton fabrics using gamma chamber. TG analysis of the treated fabrics showed a decrease of the onset decomposition temperature and increase of char formation. The burning test showed that treated fabrics burnt much slower than untreated fabrics.	[58]
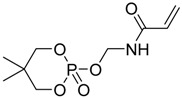	Viscose fiber fabric	The FR was applied to viscose fiber fabric through grafting polymerization. LOI values increased by increasing the grafting percentage. LOI values were almost unchanged after many washing cycles which showed the durability of the covalent bond. Cone calorimetry data of treated fabric showed a significant decrease of PHRR and THR compared to untreated fabric.	[59]
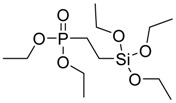	cotton	The FR was used as sol-gel precursor. It was hydrolyzed with 3-aminopropyltriethoxysilane (APTES) in deionized water in presence of HCl followed by the addition of a crosslinker. The fabrics were treated by impregnation in solutions and cured at 150 °C after processing. The burning test showed that all treated cotton fabrics burned completely with significant reduction of burning time and burning rate compared to untreated fabrics. The FR exhibited condensed phase mode of action as TG analysis showed an increase of char formation.	[60]
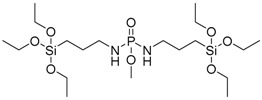	cotton	The cotton fabrics were treated by soaking in finishing baths of FR, each at different concentrations but all at pH = 5. The FR can react with cellulose without using other crosslinkers. LOI values of treated fabrics increased by increasing the FR concentration. The treated fabrics were found durable up to 50 washing cycles as no change had been noticed on the LOI values or the physical performance of the treated fabrics.	[61]
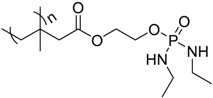	cotton	The cotton fabrics were treated by impregnation in an aqueous solution of FR, binder, crosslinker and pyrovatex. LOI values of the treated fabrics with different binders and crosslinkers were below 21 which means they can ignite easily and burn rapidly.	[44]
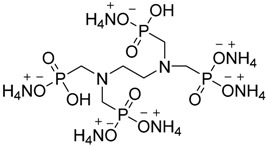	cotton	The fabrics were treated by immersion in an aqueous solution of the FR in the presence of a buffer and shrinking agent. LOI measurements showed an increase in LOI values with increases in the FR concentration. The treated fabrics showed durability up to 30 laundry cycles which was considered as semi-durable flame-resistant. The vertical burning test showed no afterflame or afterglow and a decrease in the char length with an increase in the FR concentration.	[62]
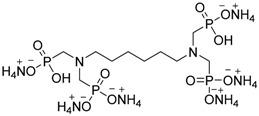	cotton	The fabrics were treated by immersion in an aqueous solution of the FR in the presence of a shrinking agent The treated fabrics showed durability up to 30 laundry cycles which was considered as semi-durable flame-resistant The vertical burning test showed no afterflame or afterglow and a decrease in the char length with an increase in the FR concentration.	[63]
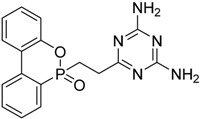	PA6 fibers	Meltable flame retardant which facilitates the compounding process. Rheological measurements showed that this FR behaves as a plasticizer for PA6. TG analysis showed higher char formation of formulated PA6 with the FR compared to neat PA6, while PCFC showed a predominant action in the gas-phase mode. UL-94 test showed a V0 rating with 15 wt % of FR in PA6.	[64]
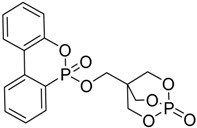	PET and PBT fibers	The UL-94 test for the formulated PET and PBT fibers with FR showing a V0 rating.	[65]

**Table 3:** Main results achieved by treating fabrics with biomacromolecules.

Type of P-based FR	Textile material	Highlights	Durability (washing fastness)	Ref.
Casein	Cotton	Anticipation of cotton degradation as assessed by TG analyses (T_10%onset_ values: 285 °C vs. 329 °C for treated and untreated fabrics, respectively). High residue (18%) at 600 °C in nitrogen (TG analyses). Significant increase of the total burning time (+40%), and strong reduction of the total burning rate (−35%) with respect to untreated cotton in flammability tests. Remarkable increase of the final residue after flammability tests (+34%). Strong reduction of TTI (−52%) and of PHRR (−27%) with respect to untreated cotton (Cone calorimetry tests).	No	[71]
Casein	Polyester	Anticipation of polyester degradation as assessed by TG analyses (T_10%onset_ values: 315 °C vs. 400 °C for treated and untreated fabrics, respectively). Increase of LOI after the biomacromolecule treatment (from 21% to 26%). High residue (22%) at 600 °C in nitrogen (TG analyses). Significant reduction of the total burning rate (−67%) with respect to untreated polyester in flammability tests. Remarkable increase of the final residue after flammability tests (+77%). In cone calorimetry tests, strong reduction of TTI (from 112 to 62 s, for untreated and treated polyester) and increase of the residue (from 2, neat polyester, to 11%, treated fabric). Melt dripping phenomena also observed for the treated fabrics.	No	[72]
Casein	Cotton-polyester blend (35%–65%)	Anticipation of fabric blend degradation as assessed by TG analyses (T_10%onset_ values: 332 °C vs. 304 °C for treated and untreated fabrics, respectively). High residue (22%) at 600 °C in nitrogen (TG analyses). Remarkable increase (+64%) of the total burning time and decrease of the total burning rate (−36%) in flammability tests. Cone calorimetry tests: strong reduction of TTI (from 30 to 12 s, for untreated and treated fabric blend) and of PHRR (−15%) and increase of the residue (from 3, neat blend, to 5%, treated fabric). Slight increase of LOI after the biomacromolecule treatment (from 19% to 21%).	No	[72]
DNA	Cotton	Anticipation of cotton degradation as assessed by TG analyses (T_10%onset_ values: 244 °C vs. 335 °C for treated and untreated fabrics, respectively). High residue (34%) at 600 °C in nitrogen (TG analyses). Self-extinction; flame out reached within 2 s. Cone calorimetry tests: no ignition under a heat flux of 35 kW/m^2^. Significant increase of LOI (from 18, untreated cotton, to 28%, DNA-treated cotton).	No	[73]
DNA	Cotton	10 wt % is the minimum biomacromolecule add-on necessary to reach the flame out of cotton. Cone calorimetry tests performed at 50 kW/m^2^: remarkable decrease of PHRR (−60%).	No	[74]
DNA	Cotton	Being equal the final dry add-on on the fabrics, low *M*_W_ DNA (100–200 bp) is more effective in enhancing the fire behavior of cotton, with respect to high *M*_W_ DNA (3000–10,000 bp), providing the highest residues after horizontal flame spread tests and self-extinction for about the 86% of the tested samples impregnated at pH = 4. Cone calorimetry tests performed at 35 kW/m^2^ on fabrics treated with low *M*_W_ DNA: reduction of THR (−36%) and of PHRR (−40%).	No	[75]
DNA	Cotton	LbL treatments with chitosan (20 bi-layers, BL) provide self-extinction to the fabric. Cone calorimetry tests performed at 35 kW/m^2^ show a significant decrease of TTI (from 39 s, for untreated cotton, to 23 s, for 20 BL-treated fabric), of PHRR (−41%) and of THR (−32%); the LbL treatment provide an increase of the residue (from 2% for untreated cotton, to 13%, for 20 BL-treated fabric).	No	[76]
